# “We Will Appreciate Each Other More After This”: Teachers' Construction of Collective and Personal Identities During Lockdown

**DOI:** 10.3389/fpsyg.2021.703404

**Published:** 2021-08-20

**Authors:** Kathryn Spicksley, Alison Kington, Maxine Watkins

**Affiliations:** ^1^School of Education, University of Worcester, Worcester, United Kingdom; ^2^School of Psychology, University of Worcester, Worcester, United Kingdom

**Keywords:** teacher identity, social identity theory, COVID-19, lockdown, remote teaching, collegiality, teacher peer relationships, discourse analysis

## Abstract

In March 2020, schools in England were closed to all but vulnerable children and the children of key workers, as part of a national effort to curb the spread of the Covid-19 virus. Many teachers were required to work from home as remote learning was implemented. Teaching is primarily a relational profession, and previous literature acknowledges that supportive relationships with peers help to maintain teachers' resilience and commitment during challenging periods. This paper reports on findings from a small-scale study conducted in England during the first national lockdown beginning in March 2020, which explored the impact of the requirement to teach remotely on teachers' identity and peer relationships. A discourse analysis, informed by the aims and practices of discursive psychology, was conducted in order to explore the association between constructions of peer support and responses to the Covid-19 pandemic. Findings indicate that teachers who presented their professional self-identity as collective rather than personal appeared to have a more positive perspective on the difficulties caused by the Covid-19 pandemic. These findings, which have implications for policymakers and school leaders, contribute to the growing field of research on the impact of the Covid-19 pandemic on education by showing the strong association between teachers' constructions of identity and their capacity to respond positively to the challenges brought about by the Covid-19 pandemic.

## Introduction

### Covid-19 and Education in England

The pandemic spread of the Covid-19 virus in 2020 created unprecedented disruption to education on a global scale. School buildings were reported to have closed in 188 countries by April 2020 (UNICEF, 2020). In England, schools closed in March 2020 except for those children considered vulnerable and children of key workers (BBC, 2020). Restrictions were imposed quickly leaving little time for teachers and schools to prepare; on the 13^th^ March, Gavin Williamson, the Secretary of State for Education, spoke to school leaders at the Association of School and College Leaders (ASCL) Conference, saying that “[i]n the overwhelming majority of situations, there is absolutely no need to close a school” (Williamson, 2020, n.p.). On the 18^th^ March, only a few days later, Williamson ordered all schools to “shut their gates [and] remain closed” from 20^th^ March (UK Parliament, 2020, n.p.). Examinations were canceled and teaching was moved online, with teachers required to educate pupils remotely from home; most pupils did not return to school until September 2020 (Ofsted, 2020). Research is beginning to detail the negative effect that this initial lockdown and subsequent disruption has had on the well-being and attainment of many pupils (Young Minds, 2020; Rose et al., 2021).

Less attention has been paid to the impact on teacher peer relationships, although there have been indications that teachers sought out supportive relationships with their colleagues in order to maintain resilience during this challenging time (Kim and Asbury, 2020; Klapproth et al., 2020), and that senior leaders reorientated their attention toward relational aspects of schooling (Ferguson et al., 2021). The requirement to teach online had an impact on pedagogy (Greenhow et al., 2020; Spoel et al., 2020; Carpenter and Dunn, 2021), attainment (Ofsted, 2020; Rose et al., 2021), student motivation (Ofsted, 2020; Zaccoletti et al., 2020), and student–teacher relationships (Jones and Kessler, 2020; Moss et al., 2020; Wong, 2020). Headteachers reported that their strategies of leadership shifted becoming more closely aligned to an ethic of care, recognizing the traumatic nature of the crisis (Beauchamp et al., 2021).

This article explores how teachers discursively constructed their relationships with peers during the first lockdown in England (March 2020), and how this impacted on their perspectives on the crisis and their construction of a professional identity. Findings show that teachers who constructed a salient *social* identity portrayed more positive perspectives on the Covid-19 crisis, whereas those who constructed a salient *personal* identity had more negative perspectives. The reasons why these teachers chose to construct their professional identities in these ways are also touched upon. We show that senior leaders used a social identity to present a positive professional identity, and that teachers who were considering leaving the profession discursively justified their loss of commitment through the foregrounding of a personal identity.

### Teachers' Mental Health: Stress and Social Support

The association between teaching and mental health difficulties is long established (Blase, 1982; Kalker, 1984; Kyriacou, 1987; Guglielmi and Tatrow, 1998), and consequently a vast literature exists documenting teacher stress and burnout. Before the Covid-19 pandemic, teachers stress was already recognized as a serious problem (Johnson and Birkeland, 2003; Johnson et al., 2005; Newberry and Allsop, 2017), causally linked to teacher burnout and attrition (Betoret, 2009; Jones and Youngs, 2012; Skaalvik and Skaalvik, 2016; Ryan et al., 2017). Teacher stress has been attributed to negative relationships with pupils (Aldrup et al., 2018; Harmsen et al., 2018), insufficient support within school or negative relationships with teaching colleagues (Troman, 2000; Van Dick and Wagner, 2001; Yuan and Lee, 2016), and accountability procedures leading to increased workload and loss of agency (Perryman, 2007; Brown and Manktelow, 2016; Towers and Maguire, 2017). The Covid-19 pandemic brought additional stressors for teachers, including Covid-19 related anxiety (Pressley, 2021) and teachers reported higher feelings of nervousness, anger, and boredom while remote teaching (Letzel et al., 2020) and on their return to school (Ozamiz-Etxebarria et al., 2020). Research conducted during the Covid-19 pandemic has, however, highlighted several factors which can mitigate teacher stress, including autonomy supportive leadership (Collie, 2021), social support (Zhou and Yao, 2020), and feelings of self-efficacy (Rabaglietti et al., 2021).

Although social support is recognized as a way of reducing stress generally (Viswesvaran et al., 1999; Ozbay et al., 2007; McKimmie et al., 2019) and specifically within education (Kinman et al., 2011; Larrivee, 2012), it is recognized that certain groups have tendencies toward particular methods of coping with stress. Strategies of developing and sustaining social support in order to alleviate stress appear to be more common amongst women rather than men (Taylor, 2011); this is in line with research which has identified maladaptive and avoidant coping strategies as more often practiced by males in response to stress, whereas females will more often use adaptive coping strategies (Gentry et al., 2007; Adasi et al., 2020). Studies have identified this gendered pattern in teachers' responses to the pandemic (Klapproth et al., 2020; Truzoli et al., 2021). As three quarters of the teaching population in England are female (Gov.uk, 2020), it would therefore be expected that developing and maintaining social support networks would be a prominent coping strategy to manage stress amongst teachers working in this context.

The present article is influenced by the field of discursive psychology (Potter and Wetherell, 1987; Edwards and Potter, 1992); as such, the focus of research is not on the causes of psychological issues such as stress, or on the efficacy of coping mechanisms used to prevent or cure such problems. Instead, discursive psychological research focuses on how people *talk* about psychological issues such as stress, and how the introduction of such issues into talk are *used* to achieve certain aims. Researchers using discursive psychological approaches identify the relationship between causal attributions of stress in the workplace (Kinman and Jones, 2005), and explore what is considered “normative” with regard to workplace stress (Harkness et al., 2007).

Such discursive approaches to education seek to identify the discursive associations and strategies which are deployed when teachers talk about their working lives. In their research with 15 Scottish secondary school teachers, Hepburn and Brown showed that in their research conversations teachers used “[s]tress as a category, and its ability to be generalized to the whole population of teachers [to] build immunity from any accusations” (2001, p. 701). Stress was called upon within research conversations to protect teachers' sense of positive professional identity and to defend them from accusations of impropriety. Kelly and Colquhoun (2010) found that reducing stress was constructed by policymakers as key to improving school improvement, with subsequent responsibility placed on school managers to manage stress amongst their workforce, and for individual teachers to position themselves as able to successfully manage stress. Thomson (2008) showed how one headteacher used the theme of stress in a radio interview to criticize government policy and justify decisions by headteachers to leave the profession. In such research, the focus is not on how stress manifests in individuals or how individuals cope with stressful situations, but instead on how the theme of stress is discursively deployed in conversations in order to support the speaker's construction of a positive professional identity.

### Social Relationships and Social Identity Theory

Our study was primarily driven by an interest in how teachers spoke about their relationships with colleagues during the Covid-19 lockdown. Positive social relationships are strongly associated with improved mental and physical health outcomes, including higher well-being and lower rates of mortality (Kawachi and Berkman, 2001; Cohen, 2004; Holt-Lundstrad and Smith, 2012; Tay et al., 2012). The “stress buffering hypothesis” (Cohen and Wills, 1985; Raffaelli et al., 2013) holds that supportive social relationships are able to provide a “buffer” to individuals during times of perceived stress and anxiety, protecting their mental and physical health. In their theoretical work on the importance of social relationships, Feeney and Collins have defined social support as “an interpersonal process with a focus on thriving” (Feeney and Collins, 2014, p. 113). Taking a lead from the seminal work of Bowlby (2005) on attachment theory, Feeney and Collins argue that supportive social relationships enable individuals to flourish, as well as being protective during challenging times. However, it is also acknowledged that close relationships which are negative can have detrimental effects (Bertera, 2005; Ibarra-Rovillard and Kuiper, 2011).

Developing supportive relationships with peers has been recognized within education literature as a necessary factor in maintaining teachers' resilience, commitment, and motivation. Day et al. (2007) argued that teacher identity was a composite of professional identity (reflecting policy and social trends), situated identity (involving relationships with others within a school context), and personal identity (generated from life beyond school). When all these composite elements were in balance, teachers were able to maintain commitment and resilience. However, when one or more of these composite elements became unbalanced and dominated by negative influences, teachers became at risk of losing motivation. During times of rapid change caused by internal or external events—such as that brought about by the Covid-19 crisis—“additional effort would need to be made by the individual in order to manage the imbalance” (Day et al., 2007, p. 108). Teachers were defined as vulnerable within this study when they were unable to “find a suitable strategy for coping with challenging situations” (2007, p. 108). The VITAE findings are highly relevant to the present study on the challenges faced by teachers in the Covid-19 crisis, as all teachers during the Covid-19 lockdown faced an imbalance in their professional and situated identities as national and school-level policies rapidly shifted.

The literature on the beneficial and protective effects of social support and a sense of belonging can be further illuminated by social identity theory. Social identity is the sense of self that is due to a person's connection to, and identification with, a significant social group, such as family, a professional group, or friends (Tajfel, 1978; Tajfel and Turner, 1979). This collective level of identity means that people define themselves in terms of *we*, as opposed to the individual sense of self, using *I*. Collective identity indicates an individual's sense of belonging within a particular social group or community, and involves firstly a “reflexive knowledge of group membership” and, secondly, an “emotional attachment or specific disposition to this belonging” (Benwell and Stokoe, 2006, p. 25). The personal self, in contrast, is a concept of the self as individual, differentiated from others (Brewer and Gardner, 1996). The social identity approach has been applied in organizational literature, exploring topics such as leadership (Steffens et al., 2014), stress (Haslam and Reicher, 2006; Muhlhaus and Bouwmeester, 2016), and motivation (Haslam et al., 2000). Haslam et al. (2000) argued that this sense of “we-ness” plays an important motivational role, while also facilitating positive and sustainable organizational outcomes.

The claim that a sense of belonging within a social group can act as a protective factor for the individual, and improve their sense of well-being, was further established by Jetten et al. (2017), who argued that identification with a meaningful social group should be considered to be the “social cure” in relation to health and well-being. Such findings are particularly pertinent to our understanding of self-identity in times of crisis. Drury (2018) argued that a shared identity leads to an *expectation* of support from others during crisis situations, which in turn leads to an increased sense of collective efficacy and well-being. This phenomenon is referred to as “collective resilience” (Drury et al., 2009; Drury, 2012), where it has been recognized that “shared social identity based on group membership can explain social support and hence coping, survival and wellbeing” (Drury, 2012, p. 210).

A sense of social identity has been found to have a positive impact on individuals during the Covid-19 crisis. Kim and Asbury found in a small-scale study of 24 teachers working in English schools that a sense of shared identity acted as a support for teachers during the Covid-19 crisis, arguing that teachers “drew upon characteristics they perceived as being widespread in the teaching profession to find ways to make remote education work for them” (2020, p. 1075). More generally, Biddlestone et al. (2020) found that collectivism positively predicted engagement with social distancing and hygiene recommendations, whereas individualism negatively predicted engagement with measures to control COVID-19.

Previous research had therefore highlighted the importance of teachers' situated identity within school contexts, and the importance of social support to teachers as a coping mechanism during time of stress (including during the Covid-19 pandemic). Our research extended previous research by specifically attending to how teachers constructed their relationships with peers during the Covid-19 pandemic lockdown, and by exploring the associations between these constructions and how psychological states (both negative and positive) were reported.

## Methodology

### Theoretical Framework and Approach to Identity

The approach taken to identity in this paper was informed by the field of discursive psychology, which seeks to “study how people deploy everyday psychological notions and manage psychological business within talk and text, and what they accomplish by such deployments” (Edwards, 2012, p. 425). Discursive psychology differs from what Edwards (2012) classifies as “scientific psychology” and is “completely different from the factors and outcomes approach that is characteristic of much mainstream social psychology” (Wiggins and Hepburn, 2007, p. 281), in that it is focused on the linguistic and interactional strategies used by individuals to construct psychological issues when involved in discursive communication—that is, through text and talk. Researchers in this field start from an understanding of language as action, rather than as representation: that is, language is not understood as a gateway to understanding pre-existing mental states, but as actively creating and defining what psychological concepts are and how they are understood (Potter, 2012).

In contrast to other psychological methods, being led by discursive psychology demands that we “begin with discourse practices” (Edwards and Stokoe, 2004, p. 499). The approach to identity and identity categories in the field of discursive psychology is informed by conversation analysis, which understands identity distinctions as constructed and used in conversation rather than as reflective of a priori groupings (Edwards, 1998). As such, analysis of identity starts from the discourse as data, rather than from categories about which the researcher has prior knowledge. How this impacts on data analysis is profound: for example, rather than comparing the responses of teachers with the responses of school leaders, an analysis of identity informed by conversation analysis would begin with data collected and look for how teachers constructed themselves as either teachers or leaders, using the conversational resources available to them. As such, the discursive deployment of pronouns during talk is particularly important in understanding individuals' identity constructions, as they reveal the groups which individuals wish to be associated with, alongside those they seek to distance their “selves” from. This approach to identity complements Davies and Harré's (1990) work on Positioning Theory and the theories of Goffman (1955); both emphasize the dynamic and fluid nature of identity in conversation. Identity is not understood as a fixed consequence of having a particular feature or background, but instead as being agentially and dynamically iterated and reiterated within discursive situations (Locher and Bolander, 2017).

The particular discursive framework employed in this research project troubled some of the assumptions of social identity theory, as established in works by Tajfel (1978) and Tajfel and Turner (1979), most obviously the claim of social psychology that groups and categories are “entities that reside in individuals and are always latently present, although they are not continuously activated” (Mieroop, 2015, p. 409). Instead, we understand identity categories as a rhetorical tool, something that individuals use in conversation to achieve certain discursive ends. As such, we recognize identity and the membership of certain identity groups as a “discursive accomplishment” (Mieroop, 2015, p. 410) or “something that is *used* in talk” (Antaki and Widdicombe, 1998, p. 2) rather than as a reflection of a group membership which exists prior to discursive construction. Social identity theory has been criticized by discursive psychologists for its presentation of identity as pre-discursive, that is cognitive and essentialist rather than constructed through language (Benwell and Stokoe, 2006). However, a number of theorists have successfully integrated the central tenets of social identity theory within a more discursive framework (Hogg et al., 1995; Mieroop, 2015; Rich et al., 2017).

In terms of social identity, therefore, researching through the lens of discursive psychology turned our attention toward the discursive ways in which individuals structure and construct their group membership. We consider identity as dynamic, actively constructed *through* talk; our interest is the discursive patterns and relationships which emerge when teachers talk about their identity and social identity categorizations. We recognize that “identity is a site of permanent struggle for everyone” (Maclure, 1993, p. 311) and that through a careful analysis of language, we are able to better pinpoint the identity work undertaken by teachers during the Covid-19 lockdown.

### Sampling and Participants

Open-ended qualitative interviews, with 30 teachers working in primary and secondary schools across England, were used to gather data for the research project. In using open-ended interviews, this research project was aligned with previous research in the tradition of discursive psychology (Lawes, 1999; Potter and Hepburn, 2005), which is distinct from other forms of discourse analysis in utilizing open ended interviews, rather than naturalistic sources, to gather data (Hepburn and Wiggins, 2007). Such interviews are sometimes referred to as “conversational” or “semi-structured” (Potter and Hepburn, 2005, p. 283), and the freedom afforded to research participants during open-ended interviews enables researchers to study their responses as actions, discursive attempts to construct specific identities, and ways of perceiving the world. Interviews explored specific aspects of remote educating and teacher peer relationships, including:

- changes to role since the partial closure of schools;
- benefits to professional relationships, family dynamics, shared activities, and enhanced learning opportunities;
- challenges of peer relationships, stress, well-being, family dynamics, physical space, work-school balance, and resources;
- influence of remote working on well-being;
- Support given during the lockdown period from peers and school leadership; and,
- Strategies for dealing with remote teaching.

Interview questions were designed to encourage participants to share their perceptions of relationships with other teachers, interpersonal dynamics, and communication. Some questions were designed to elucidate narratives from the participants about how their responses to the pandemic and their relationships with others had changed over the course of the lockdown, recognizing that “narratives and stories are vital parts of an individual's and organization's sensemaking apparatus” (Gabriel, 2015, p. 276); others were designed to encourage participants to engage in “intergroup positioning” which is “fundamentally achieved through the use of linguistic devices such as ‘we’, ‘they’, ‘us’, ‘them’, ‘I’” (Tan and Moghaddam, 1999, p. 183).

There were also practical concerns which rendered remote, individual interviews the most suitable qualitative data collection tool during the particular time in which the research was being conducted, when social distancing measures were being enforced. As a result of measures brought in to reduce the spread of the Covid-19 virus, other prominent qualitative research methods which may otherwise have been considered—such as ethnographic methods, case studies and observations—were unsuitable for this research project.

Interviews were conducted online via Microsoft Teams with each teacher and lasted between 30 and 90 min. Adopting this approach enabled the participant and a single researcher, who carried out all interviews, to see each other, building a rapport prior to the interview itself. All interviews were recorded using the facility on Teams and then transcribed. Participant names were not used; rather a unique code chosen by each teacher was added to the transcripts, providing anonymity.

The speed with which policies on Coronavirus restrictions changed in England during March 2020 meant that, as researchers wishing to catch the perspectives of teachers at this unique moment, we were required to act extremely quickly. As such, we acknowledge that in our efforts to quickly recruit participants in order to gain rapid insights into the impact of school closures on teachers in England, we employed methods of “convenience sampling” (Robson, 2011) which would not be necessary during a research project with a more conventional trajectory. Initially, personal contacts were contacted to raise awareness of the project, and this was followed by a snowball sampling strategy to achieve the required number of participant teachers for meaningful analysis. This small-scale participant recruitment target was guided by previous studies which had a similar methodological approach (Mieroop, 2005; Fest, 2015). We were aware that employing a research design that involved the recruitment of large numbers of participants may slow the research process, and in doing so prevent us from accessing data on the immediate perspectives and concerns of teachers during the first few weeks of the lockdown in England. Our study, which collected rich qualitative data from a small sample, was aligned with a number of other small-scale educational studies conducted during the early stages of the Covid-19 crisis (Anderson et al., 2020; Kim and Asbury, 2020; Sequeira and Dacey, 2020; Ferguson et al., 2021).

A sample of 30 participants was achieved (Table 1). Potential participants were sent an email inviting them to take part in the research, which also included a participant information sheet outlining key aspects of the research such as purpose, proposed schedule, time commitment, data use, and ethical issues. They were also sent a consent form outlining issues related to confidentiality and anonymity, right to withdraw, avoidance of harm, data storage and disposal, and publication of material. Those willing to participate were asked to sign and return the consent form to the researcher team by email.

**Table 1:** Characteristics of sample.

	**School phase**			**Gender**			**Career phase**			**Leadership responsibility**	
	***N***	**%**		***N***	**%**		***N***	**%**		***N***	**%**
Primary	16	53	Male	12	40	0–7 years	4	13	Leader	10	33
			Female	4	13	8–15 years	10	33	Non-leader	6	20
						16+ years	2	7			
Secondary	14	47	Male	5	17	0–7 years	4	13	Leader	9	30
			Female	9	30	8–15 years	4	13	Non-leader	5	17
						16+ years	6	20			
Total	30	100		30	100		30	100		30	100

The teacher participants (13 female, 17 male) all worked in different schools across England. The sample was made up of 16 primary and 14 secondary practitioners. Those who taught in the secondary phase taught a variety of subjects including core subjects (mathematics, English, science) and foundation subjects (art, history, geography, and modern foreign languages). Teachers were in differing phases of their careers, including eight teachers with fewer than 8 years of experience, 14 teachers with between 8 and 15 years of experience and eight teachers with more than 16 years of teaching experience^1^. Ten primary school teachers and nine secondary school teachers had leadership responsibilities under normal teaching conditions. The participants recruited for this research project were not representative of the wider teacher population, which is a limitation of the study caused by the strategy of convenience sampling. For example, whereas 24% of state employed teachers in England are male (Gov.uk, 2020), 57% of the teachers who participated in this research were male. Although a convenience sample, efforts were made to recruit participants from a range of school types, including those in rural (*n* = 7), suburban (*n* = 14), and urban (*n* = 9) settings. Again, we make no claim that the participants recruited for this project are representative of the wider school population in England.

### Data Analysis

The research questions which led the study were informed by the preoccupations of discursive psychology, and were:

RQ1 How did primary and secondary teachers in England use language to construct their psychological experiences of remote teaching during the Covid-19 lockdown?
RQ2 How did teachers discursively construct their relationships with other teachers while remote teaching during the Covid-19 lockdown?
RQ3 How did the construction of social relationships during the Covid-19 lockdown function discursively to justify particular responses or actions by teachers?

**Figure 1:**
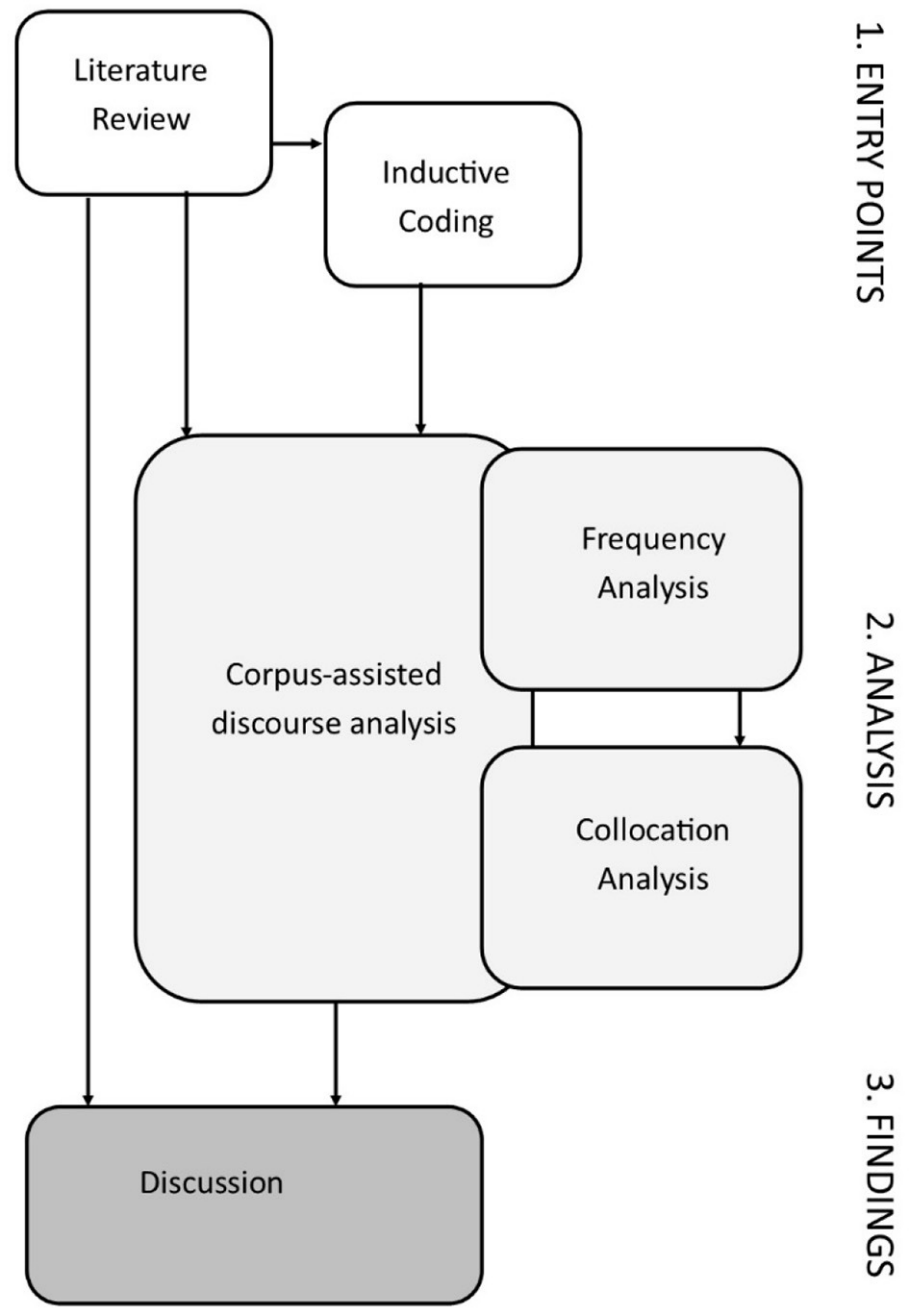
Stages of research analysis.

Analysis of data took place in several stages, as shown in Figure 1. In line with much research in discursive psychology, the data was initially coded to identify emergent themes and linguistic patterns, in a “precursor to the analysis [which involved] sifting through the larger data corpus for instances of a phenomenon” (Wiggins and Potter, 2007, p. 84); the findings from this initial coding and the literature review were used as “entry or starting points” (Baker et al., 2008, p. 295) into the corpus of interview data. Following the identification of interesting linguistic features, a corpus-assisted discourse analysis was then performed to verify the extent of these features and to explore them in greater detail. As such, analysis of the interviews involved an integration of inductive coding and corpus linguistic methods, involving an “interdisciplinary application of methods” (Fest, 2015, p. 49). Corpus linguistics is defined as a “scientific method of language analysis [which] requires the analyst to provide empirical evidence in the form of data drawn from language corpora in support of any statement made about language” (Brezina, 2018, p. 2); usually, it involves the use of specialized computer software to identify linguistic patterns within a body of texts selected by the researcher. The use of corpus linguistic methods to isolate discursive strategies of identity construction is well-established within linguistic research (Baker, 2006; Bednarek and Martin, 2011; Bakar, 2014; Fuoli, 2018). There is no prescribed method for conducting analysis which combines thematic and corpus approaches, as corpus linguistics is an emergent method within education research (Pérez-Paredes, 2021).

During analysis, we attended to participants' attempts at self-categorization, rather than starting from a priori identity groupings such as age, gender, ethnicity, leadership status, or length of service. That is, we were more interested in how participants constructed their own identity, than in pre-existing identity categories that we as researchers may attach to them (Fitzgerald, 2012; Paulsen, 2018). At the initial stage of coding, the discursive utilization of pronouns was identified as a phenomenon present within the corpus which merited further investigation. Teachers who constructed a collective identity with their teaching peers through the use of the pronoun *we* appeared to have a more positive perspective on the experience of remote teaching during the Covid-19 lockdown than teachers who constructed a salient personal identity, using the pronoun *I* to foreground their individual concerns. The use of personal pronouns, such as *I* and *we*, “gives a sense of whom a speaker identifies himself with” (Lenard, 2016, p. 166), and are used to stand in for membership categories which have been previously introduced by speakers during conversations, as Sacks explains:

“*If you've used any membership categorization device category, i.e., any category like male-female […] you can on some next occasion wherein you want to refer to the same object, use a pronoun to do it. If you've referred to a category in its plural form, e.g., […] women, then you choose from a plural pronoun, most particularly ‘we’ or ‘they,’ and you may pick ‘we’ or ‘they’ by reference to whether you are, or propose to be, a member of that category.”* (Sacks, 1992, p. 334).

The use of *we* indicates an attempt to construct or maintain an association *with* the group, whereas the use of *they* an attempt by the speaker to distance himself *from* the group. Individuals present their affiliation with an institution by using personal pronouns (Drew and Sorjonen, 1997), and teachers' pronoun choice can indicate the extent to which they claim alignment with their school (Spicksley and Watkins, 2020).

At the following stage of “analysis” (Wiggins and Potter, 2007) we employed a corpus-assisted discourse analysis to explore the phenomenon of interpersonal pronoun use in more depth (Hepburn and Potter, 2003; Pérez-Paredes, 2021). At this stage, as we moved away from initial coding and toward analysis, “simple counts” of the pronouns *I* and *we* were used as an “aid to understanding the patterning” (Hepburn and Potter, 2003, p.189) of pronoun use. This stage of analysis was oriented around the hypothesis that teachers who foregrounded the pronoun *we* had a more positive perspective on remote teaching during the Covid-19 lockdown than teachers who foregrounded the pronoun *I*. The research questions for this stage of analysis were therefore as follows:

RQ1 Is there a pattern of pronoun usage (we/I) across the corpus of interviews?
RQ2 If evident, is this pattern predictive of positive or negative constructions of the experience of remote teaching during the Covid-19 lockdown?

A recognized analytical technique in corpus linguistics is the use of quantitative data to isolate representative cases, which are then subject to further qualitative analysis (Mieroop, 2005; Bednarek, 2011). In the present study, quantitative data on pronoun use was employed to identify two sub-corpora: one in which the participants foregrounded the use of “we,” and one in which the use of “I” was foregrounded. Further analysis using both quantitative and qualitative methods was then used to compare these contrasting sub-corpora in response to the research questions.

Corpus linguistic analysis of data is facilitated through specialized computer programs. In this project, analysis of pronoun use within the interview data was undertaken using Wordsmith 7.0 (Scott, 2016), which facilitated the construction of wordlists (frequency counts of words within a specific corpus) and concordances (which show all the occurrences of a target word within their context, to reveal linguistic patterns and associations). Corpus linguistics is a comparatively “young discipline that is […] witnessing a rich debate in terms of methodological foundations” (Pérez-Paredes, 2021, p. 35). Although corpus linguistic methods are traditionally associated with the macro-analysis of large data sets (McEnery and Wilson, 2001; Baker, 2006), such techniques can be effectively used to isolate patterns of language in smaller data sets at a meso-level, or even in individual texts (Bednarek, 2011). One of the advantages of incorporating corpus methods into a discourse analysis is to reduce researcher bias, improving the validity and reliability of findings by introducing a quantitative aspect to the research (Baker, 2006, 2012; Mautner, 2009). However, within critical fields which employ discourse analysis as a research method (such as discursive psychology) there is a resistance to seeking neutral objectivity and instead a recognition that “bias is unavoidable when conducting social research” (Baker, 2012, p. 255), and even the selection of which numbers are investigated is a subjective decision, driven by the research question and researcher interest and knowledge.

Corpus linguistic approaches have been successfully used in previous research to better understand the use of pronouns in constructing educational and institutional identities, within relatively small collections of spoken data. Fest (2015) first conducted a qualitative thematic analysis on 14 interviews with students concerning an online assessment tool, before subjecting these interviews to a corpus linguistic analysis which began by analyzing the frequency of pronoun usage. Mieroop's (2005) research took the opposite approach, beginning with a quantitative analysis of pronoun usage within a corpus of 40 speeches. This quantitative analysis enabled Mieroop to isolate a sub-corpus of speeches in which the speaker presented with a strong institutional identity; this sub-corpus was then subject to a further qualitative analysis to isolate the particular strategies employed by these speakers to construct a strong institutional identity through discourse. Combining qualitative and quantitative data by synthesizing traditional thematic approaches to data analysis and corpus linguistic methods enables “both an in-depth view and an overview of the corpus” (Mieroop, 2005, p. 108) while facilitating researchers to gain “new insights into the data” (Fest, 2015, p. 64).

The overall methodological approach therefore recognized an alignment between social identity theory and discursive approaches to the interpretation and analysis of data (Rich et al., 2017), and was located with a long history of education research which has explored how teachers construct their professional identities through discourse (Maclure, 1993; Alsup, 2005, 2019; Urzúa and Vásquez, 2008; Bates, 2016). In such research, it is recognized that “motive talk […] does not have a simple inner referent but is a performative speech act in a complex language game” (Edwards and Potter, 1992, p. 141). The focus of analysis was on how teachers constructed and presented their identities through the linguistic affordances offered through their semi-structured interviews, and the effects that these constructions achieved (Fairclough, 1992; Benwell and Stokoe, 2006; Zhang Waring, 2018). Corpus linguistic methods supported the theoretical decision to focus on the identities that teacher participants chose to actively construct for themselves through discourse.

### Research Ethics

This study was reviewed and approved by the University's Arts, Humanities, and Education Research Ethics Panel, and ethical guidance from the British Educational Research Association (BERA, 2018) and the University were followed throughout the study. Signed consent forms were required from all participants, and if a teacher wished to withdraw from the study, they were able to contact the research team and request this without explanation. All data were stored and destroyed in accordance with University policy, GDPR (2018) and the Data Protection Act (2018a,b) (ICO, 2021).

## Findings

### Initial Coding

A number of accounts of teaching remotely suggested that teachers perceived themselves to be working with their colleagues in a collective effort. These teachers' narratives constructed the experience of remote teaching as a shared endeavor:

*We're sharing all the lesson plans and ideas for lessons. We're all doing the tutorials and sharing information about the students when necessary. We have a department meeting each Monday evening and then a message from the Head each Monday morning. We're a social department and so are used to communicating all the time and that has leaked into the weekends with some interaction*. (Jenny)

*We're more than just a department of individual teachers, we're a solid team who work well together, respect each other, learn from each other, and support each other*. (Tamara)

*I think we've stayed strong as a school, shared our expertise and remained confident in our ability to do the job we trained for […] even in these strange times*. (Noah)

During these utterances, use of the pronoun *we* constructed a sense of collegiality in schools; for the individuals, the use of the pronoun *we* served a particular function in the discursive construction of identity. By constructing their selves as being part of a wider collective team, these teachers were able to tacitly position themselves as having particular personality traits which are generally considered to be positive. These traits include sharing and communicating effectively, being social, respecting others, and teaching confidently. For these teachers, constructing a sense of social identity was a way of rhetorically positioning themselves as having valuable characteristics.

In contrast, other participants argued that they felt disconnected from their colleagues and missed the day-to-day support they had previously received in school:

*The main change has been that there is no-one to discuss lesson plans with. That sense of support has disappeared, not intentionally, but the reality is that we are dealing with everything on our own now […] It's very lonely. I actually miss staff meetings*. (Peter)

*I'm far more detached now as I'm not hearing about all the things that would usually be happening around school. We're completely cut off and that's hard to deal with*. (Camilla)

The utterances of Peter and Camilla involved a “shift of footing” (Goffman, 1981) as pronoun use changed from *I* to *we*. The function of these utterances is to justify or explain why Peter and Camilla are experiencing the negative emotion of loneliness. Camilla and Peter use the pronoun *I* to emphasize their isolation from colleagues, alongside *we* to construct this not as an individual problem which only affects them, but also a problem experienced by others within their setting. Pronoun choice enables Camilla and Peter not only to emphasize their isolation from colleagues, but also to construct their feelings of isolation as normal and as being experienced by others, lessening the possibility of them being perceived as dysfunctional or antisocial.

For some participants, constructing a sense of isolation functioned as an explanation for decisions to leave teaching. Pronoun use in such cases was again found to be significant. Susan and Robert used *I* to emphasize their sense of individualism during the crisis:

*Yes, it's been very stressful from a professional point of view and a personal one. Professionally, I've found it hard to be isolated from the others and feel as if I'm missing out on things […]it's starting to affect the bond I used to feel with being a teacher*. (Susan)

*I'm thinking about leaving the profession, definitely about leaving the school at least. This has given me time to think about it without having to be with them every day*. (Robert)

The decision to leave teaching is often associated with a sense of failure (Smith and Ulvik, 2017). In these utterances, when teachers constructed a professional identity which was faltering or at risk, use of the pronoun *I* functioned to emphasize their feelings of isolation from their colleagues. By emphasizing how they felt separated from their school community, Susan and Robert sought to excuse and make acceptable the decision to leave teaching, which is often associated with negative traits such as a lack of commitment or resilience.

Initial coding had therefore indicated that choices about pronoun use were one important way in which participants discursively navigated the complexities of reflecting on the difficulties caused by the Covid-19 pandemic and the requirement to teach remotely. Furthermore, whereas the use of *we* and the construction of a *collective* identity within the participants' schools appeared to be associated with positive perspectives on the Covid-19 crisis, the use of *I* and the foregrounding of a salient *personal* identity appeared to be associated with negative perspectives and emotional responses.

### Discourse Analysis

#### Collective Identity and Personal Identity Groupings

Using Wordsmith, it was possible to identify the frequency of the pronouns *I* and *we* within each interview transcript, and (for comparative purposes) across the entirety of the interviews. The identification of these differing “person deictics” (Mieroop, 2015, p. 414) provided an innovative way in to exploring the identity constructions of research participants, indicating each participant's sense of “we-ness” within their school community (Haslam et al., 2000).

Results from the analysis of all interview transcripts indicated that there were indeed significant differences in the use of the pronouns *we* and *I* across interview transcripts (Table 2). Five participants (Noah, Maria, Isaac, Ivy, and Edwin, henceforth referred to as the “CI Group”) used *we* more frequently than *I* in their responses, indicating the construction of a salient *collective* identity. Twenty-five participants used *I* more frequently than *we*; considering the private and individualized nature of the semi-structured interviews conducted, this overall preference for the pronoun *I* across the dataset was to be expected. Of these 25 participants, however, five participants (Ava, Tamara, Matilda, Timothy, and Christopher, henceforth, the “PI Group”) had a significant preference for the pronoun *I* over *we*, indicating the construction of a salient *personal* identity. The PI group was formed of participants who displayed more than a 3% difference between their use of the pronoun *we* and their use of the pronoun *I*.

**Table 2:** Interpersonal pronoun data across sample.

**PSEUDONYM**	**I freq**	**I %**	**WE freq**	**WE%**	**Difference I/WE Freq**	**Difference I/WE %**	**Analysis sub-group**
Isaac	40	2.15	44	2.37	−4	−0.22	CI
Noah	30	1.82	31	1.88	−1	−0.06	CI
Maria	32	1.84	33	1.9	−1	−0.06	CI
Ivy	37	2.14	38	2.19	−1	−0.05	CI
Edwin	42	2.23	43	2.28	−1	−0.05	CI
Steve	50	2.63	48	2.53	2	0.1	None
Paul	38	2.47	33	2.15	5	0.32	None
Grace	40	2.65	27	1.79	13	0.86	None
Camilla	36	2.78	22	1.7	14	1.08	None
Peter	84	4.32	43	3.09	41	1.23	None
Gary	47	2.82	26	1.56	21	1.26	None
Oliver	42	2.65	20	1.26	22	1.39	None
Helen	35	2.67	15	1.14	20	1.53	None
Harry	48	3.51	24	1.76	24	1.75	None
Audrey	39	2.76	13	0.92	26	1.84	None
Jenny	51	3.09	15	0.91	36	2.18	None
Aiden	45	3.46	14	1.08	31	2.38	None
Susan	67	3.5	19	0.99	48	2.51	None
Mark	58	3.89	20	1.34	38	2.55	None
Lily	49	3.78	15	1.16	34	2.62	None
Robert	29	3.05	4	0.42	25	2.63	None
Alexander	52	3.54	13	0.88	39	2.66	None
Sally	75	4.33	28	1.61	47	2.72	None
Ethan	59	4.23	20	1.43	39	2.8	None
Hayden	134	4.44	44	1.46	90	2.98	None
Ava	70	4.88	21	1.46	49	3.42	PI
Matilda	64	4.48	12	0.84	52	3.64	PI
Timothy	84	4.32	13	0.67	71	3.65	PI
Christopher	87	4.48	16	0.82	71	3.66	PI
Tamara	56	4.35	8	0.62	48	3.73	PI
Across all interviews	1,586	3.3	722	1.5	864	1.8	

There were some noticeable similarities between the teachers within the CI Group. All CI Group participants were experienced teachers, with more than 8 years of experience and having a leadership role. Four of the five were teaching within primary schools, with only one (Maria) teaching within a secondary setting. The gender of teachers within the CI group was, however, quite balanced, with three male and two female participants within this category. In terms of the PI Group, there was one noticeable pattern which emerged in terms of characteristics. Four PI teachers worked in secondary settings and one in primary, reversing the trend seen within the CI Group. In terms of the other characteristics, the PI Group had a wider spread of teachers from all career phases than the CI Group. Three of the teachers in the PI group had leadership roles, and two were non-leaders, again indicating a wider spread of characteristics than the CI Group in which all teachers identified as leaders. Like the CI Group, gender was quite balanced, including three female and two male teachers.

**Figure 2:**
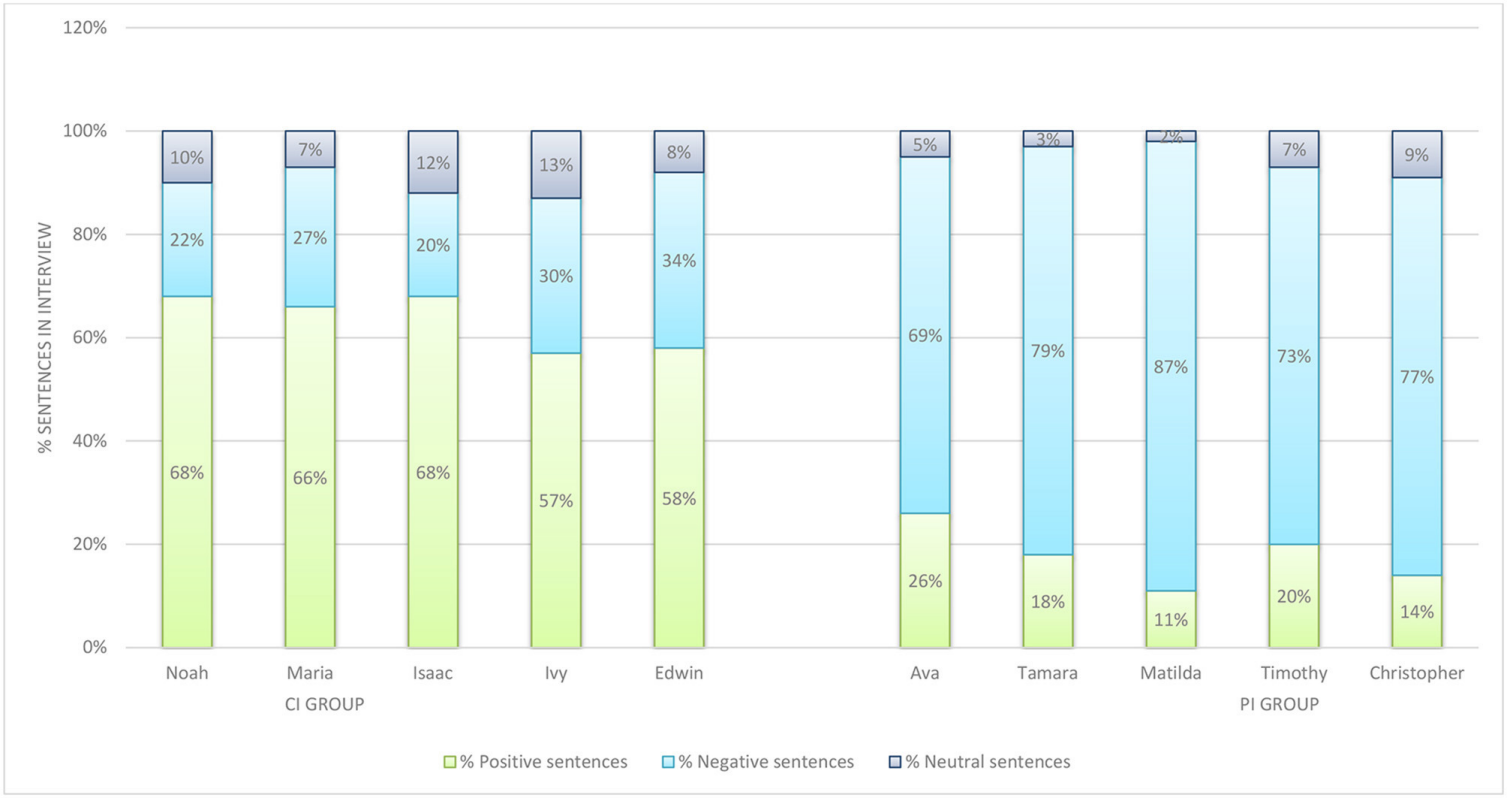
Comparative analysis of CI and PI Group utterances.

Collective Identity Group interviews and PI Group interviews were then subjected to a further manual discourse analysis in order to determine these teachers' perspectives on the Covid-19 pandemic lockdown and its effects. This manual analysis was conducted to determine whether there was a significant difference between the way that teachers with a salient collective identity (CI Group) constructed the experience of teaching remotely during the Covid-19 lockdown, in comparison with teachers who had a salient personal identity (PI Group). During this discourse analysis, each sentence was evaluated as being either a positive, a negative, or a neutral utterance (Liebrecht et al., 2019). Figure 2 compares the percentage of sentences considered to be negative, positive, and neutral utterances in the interview transcripts of the CI and PI Groups, in order to enable a comparison between the perspectives of the two groups.

The PI group had a significantly higher percentage of negative utterances in their interviews, ranging from 69 to 77% of the sentences recorded in their interviews being negative. In comparison, the percentage of sentences considered negative within the CI Group's interviews ranged from 20 to 34%. The pattern was reversed with the percentage of positive utterances. In the PI Group, positive utterances as a percentage ranged from 11 to 26% of their interview transcripts. However, participants in the CI Group used positive utterances between 57 and 68% of their interview. Findings clearly showed, therefore, that members of the CI Group constructed the challenges of the Covid-19 pandemic using more positive language than members of the PI Group. Teachers who constructed a collective identity for themselves, having a preference for the pronoun *we*, constructed a more positive perspective on the Covid-19 crisis. Teachers who identified primarily as an individual, preferring the pronoun *I*, instead constructed a more negative perspective during the Covid-19 pandemic lockdown. As the findings from this discourse analysis appeared to be significant, further analysis on the difference between the discourse of CI Group and PI Group participants was then conducted, using methods commonly associated with corpus linguistics. This included further frequency analysis, and collocation analysis using concordances (which show every occurrence of a target word in context).

#### Wordlist Data

A comparison of the 20 most frequent content words^2^ across CI and PI Group interviews can be found in Table 3. There were many similarities across the groups. Both CI and PI Group participants had a high frequency of words related to their job as teachers, including *work, school, teaching*, and *teacher*. Some differences across the CI and PI Groups can be attributed to the differential between the groups in terms of phases taught: whereas *department* features as a frequent content word in the PI Group interviews, it was not present in the 20 most frequent words of the CI Group participants. As the majority of participants in the CI Group were primary teachers, and primary schools are generally not split into departments, this could explain this discrepancy. In secondary schools, which most of the PI Group worked in, work is more often organized through departments, explaining why PI Group data featured this word more prominently. The same could be true of the inclusion of *children* within CI Group data, mainly consisting of primary teachers: *students* would be a more prominent term within secondary settings, which had a higher frequency within the PI Group.

**Table 3:** Comparative analysis of 20 most frequent function words in CI and PI Group interviews.

**Function word ranking in group**	**PI Group**		**CI Group**	
	**Content word**	**Frequency in PI g roup interviews**	**Content word**	**Frequency in CI group interviews**
1	WORK	48	THINK	61
2	THINK	43	WORK	61
3	STUDENTS	33	SCHOOL	58
4	SCHOOL	32	CHILDREN	49
5	THINGS	29	STAFF	29
6	GET	28	THINGS	29
7	TIME	25	TEACHERS	27
8	FEEL	23	WORKING	24
9	TEACHING	22	SCHOOLS	23
10	TEACHER	21	SUPPORT	23
11	SUPPORT	18	TEACHING	22
12	TEAM	18	YEAR	22
13	COLLEAGUES	17	HOME	21
14	GOOD	16	TOGETHER	21
15	BACK	15	FEEL	20
16	LIFE	15	PARENTS	20
17	WORKING	15	DIFFERENT	18
18	DAY	14	MAKE	18
19	DEPARTMENT	14	WEEK	18
20	FACT	14	GROUP	16

Two content words were prominent in both CI and PI Group data: *support* and *feel*. Support was the 11th most frequent content word for PI Group teachers (*n* = 18), and the 10th for CI Group teachers (*n* = 23), indicating that both groups of teachers sought to foreground discourse around support. Further concordance analysis, detailed below, was therefore undertaken to determine whether there were any differences in the way that CI Group and PI Group teachers constructed support. The word *feel* was used a similar number of times by both CI Group (*n* = 20) and PI Group (*n* = 23) teachers, indicating that both groups wished to talk about their inner emotional or psychological states. However, it was interesting to note that *feel* was a comparatively more frequent content word used PI Group teachers, being the eighth most common content word used within this group (in comparison to being the 15th most frequent content word used by CI Group teachers). Such discourse around feelings is of particular interest within discursive psychology so, as with *support*, references to feelings were subject to further contextual analysis using concordance lines.

One interesting difference between CI Group and PI Group participants was the use of the singular or plural when using the word *teacher*. Whereas, PI Group participants foregrounded the singular *teacher* (*n* = 21), CI Group participants foregrounded the plural *teachers* (*n* = 27). This again indicates a more collective, social identity on the part of CI Group teachers, and a more personal identity being constructed by PI Group teachers. As evaluative terms associated with *teacher* could indicate the construction of a specific teacher identity by participants, *teacher* was also subject to further concordance analysis.

#### Concordance Data

##### Support

**Figure 3:**
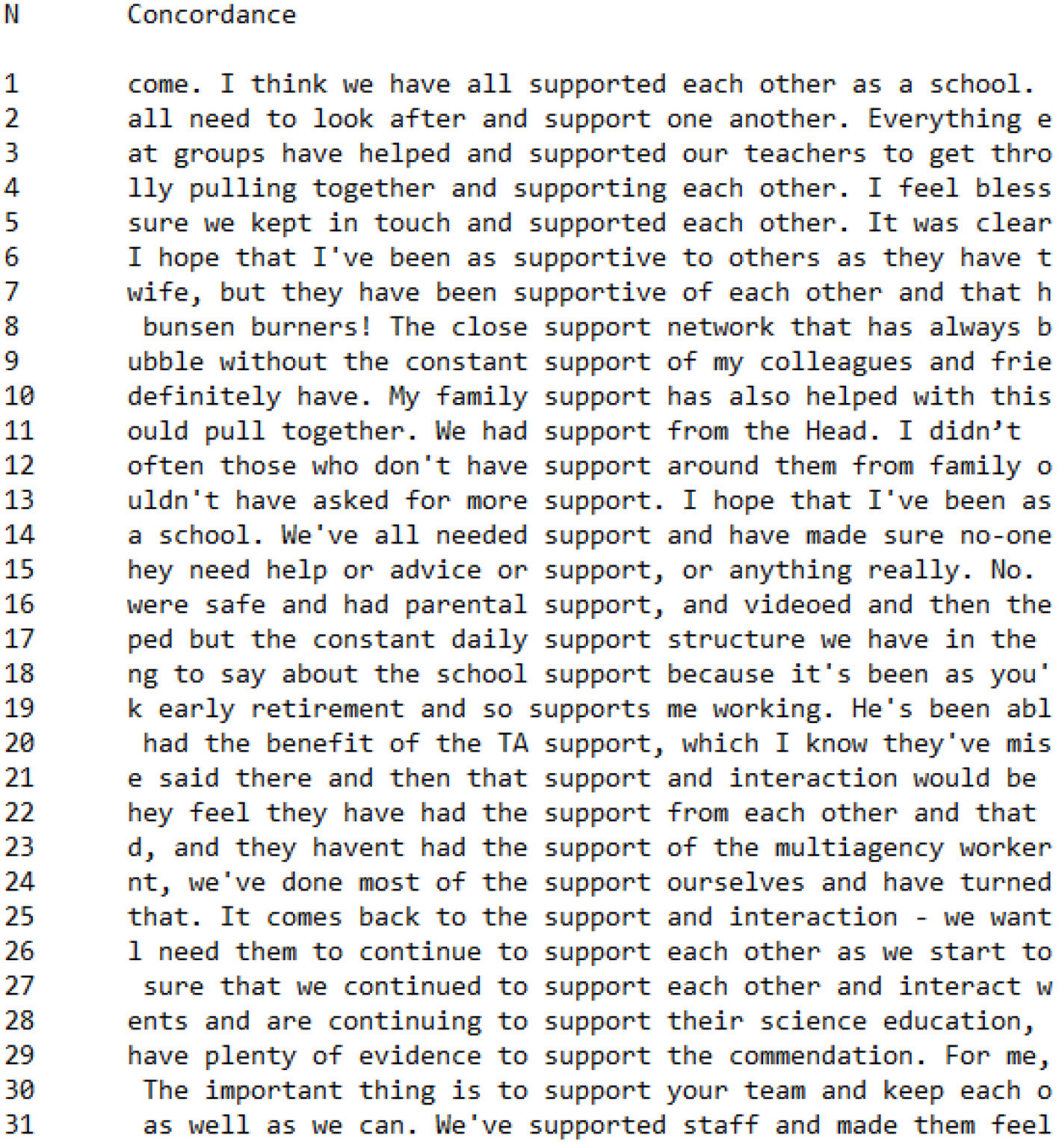
Concordance showing utterances of support* in CI Group interviews.

**Figure 4:**
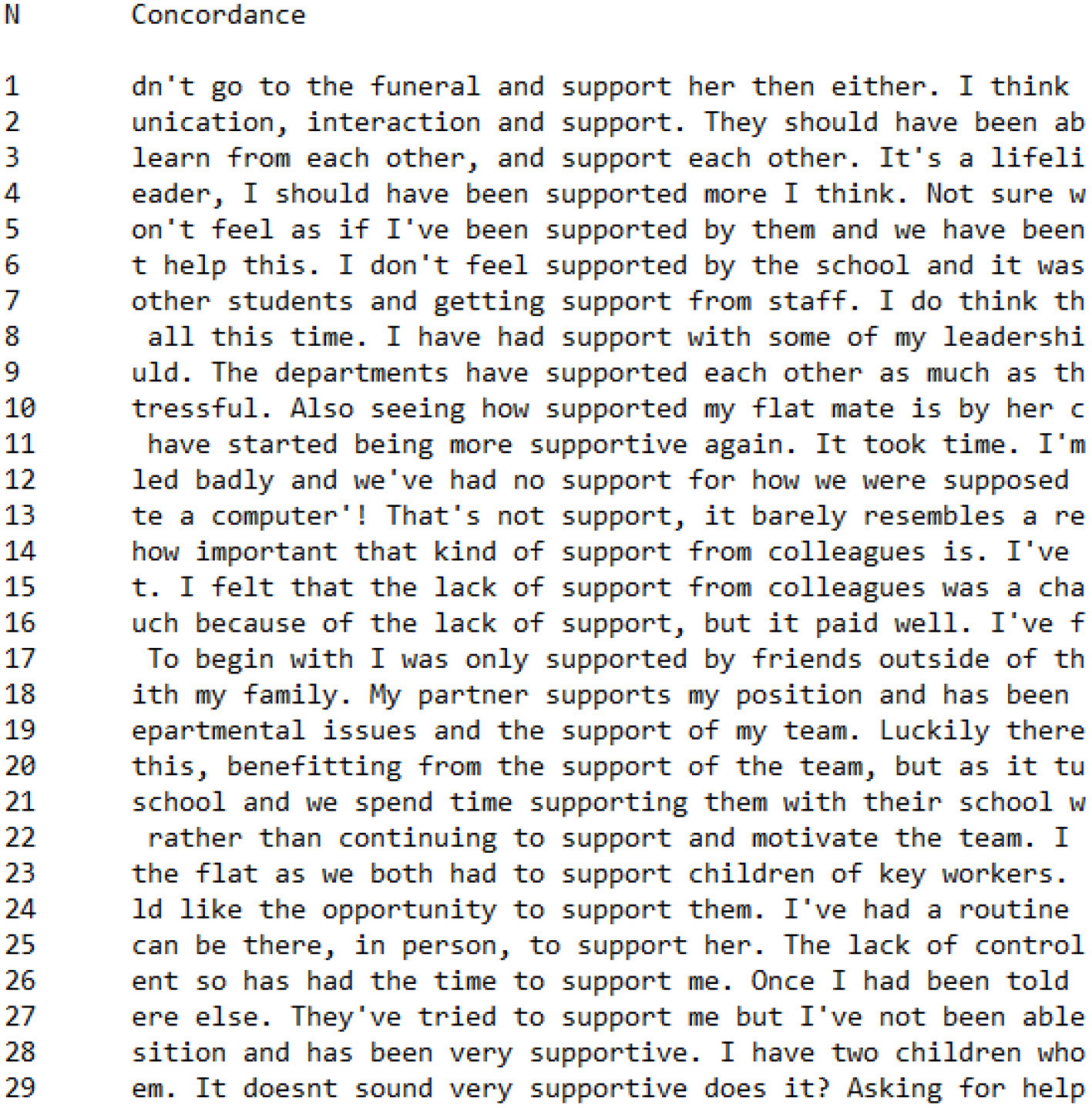
Concordance showing utterances of support* in PI Group interviews.

Figures 3, 4 are concordances which detail every occurrence of words with the root support^*^ (*support, supports, supported, supporting, supportive*) in CI (Figure 3) and PI (Figure 4) Groups. In both groups, support^*^ occurred at the same general frequency, with 31 occurrences in the CI Group and 29 occurrences in the PI Group interviews. This similarity in frequency suggests that both teachers who had a salient collective identity and teachers who had a salient personal identity worked to discursively construct support as an important factor in their presentation of teaching during the Covid-19 pandemic lockdown.

Two noticeable discursive patterns were evident in the CI Group data regarding associations with the word *support*. The first was a temporal construction of support as continuing or ongoing, as in the following concordance lines. Support was twice described as constant:

without the **constant support** of my colleagues
**constant** daily **support** structure we have

There was also a pattern through which support was constructed as a continuing process:

we **continued** to **support** each other
I need them to **continue** to **support** each other
**continuing** to **support** their science education

Within the CI Group, therefore, support was constructed as something which was ongoing and reliable, with a tacit construction of support during the Covid-19 pandemic as a continuation of support prior to these difficulties.

Second, the word support was discursively associated with the collocation *each other* (*n* = 7) or one *another* (*n* = 1), as in the following statements:

we have all **supported each other**
kept in touch and **supported each other**
been **supportive** of **each other**
look after and **support one another**

In these utterances, *support* is constructed as a communal and collegial enterprise: the term support indicates a process through which all members of a group are involved in supporting and being supported simultaneously.

In PI Group interviews, the most noticeable pattern is associations which give the impression of a support deficit (*n* = 10), as in the following statements:

**don't** feel as if I've been **supported** by them
I **don't** feel **supported** by the school
We've had **no support**
I felt that the **lack of support**
I **should** have been **supported more**

In contrast to the CI Group—in which participants made efforts to construct support as a shared, communal process—in these PI Group utterances there was again a focus on the individual, indicated by the close association of *I* and *me* with constructions of support (*n* = 15):

**I** felt that the lack of **support**
has had the time to **support me**
**I** was only **supported** by friends

Even when support was not constructed as deficient by PI Group participants, this construction of support as being focused on the individual remained:

**I** have had **support**
They've tried to **support me**

There appeared, therefore, to be a difference in the way that PI Group and CI Group teachers talked about support. For CI Group teachers, support was constructed as a communal activity, shared by everyone. In contrast, for PI Group teachers—who had a salient personal identity—support was constructed as something given to an individual by others, in an almost transactional process.

##### Teacher Identity

**Figure 5:**
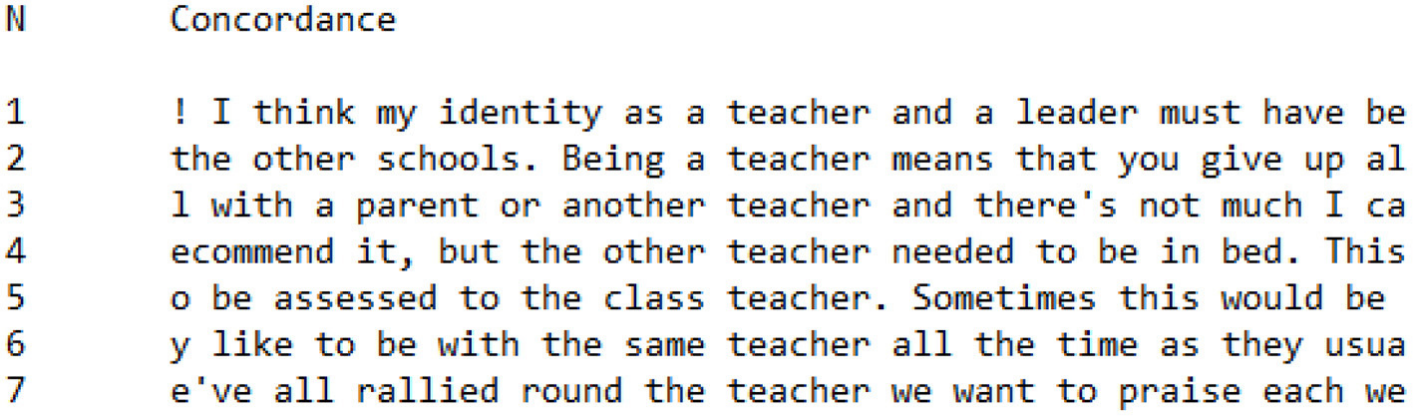
Concordance showing utterances of *teacher* in CI Group interviews.

**Figure 6:**
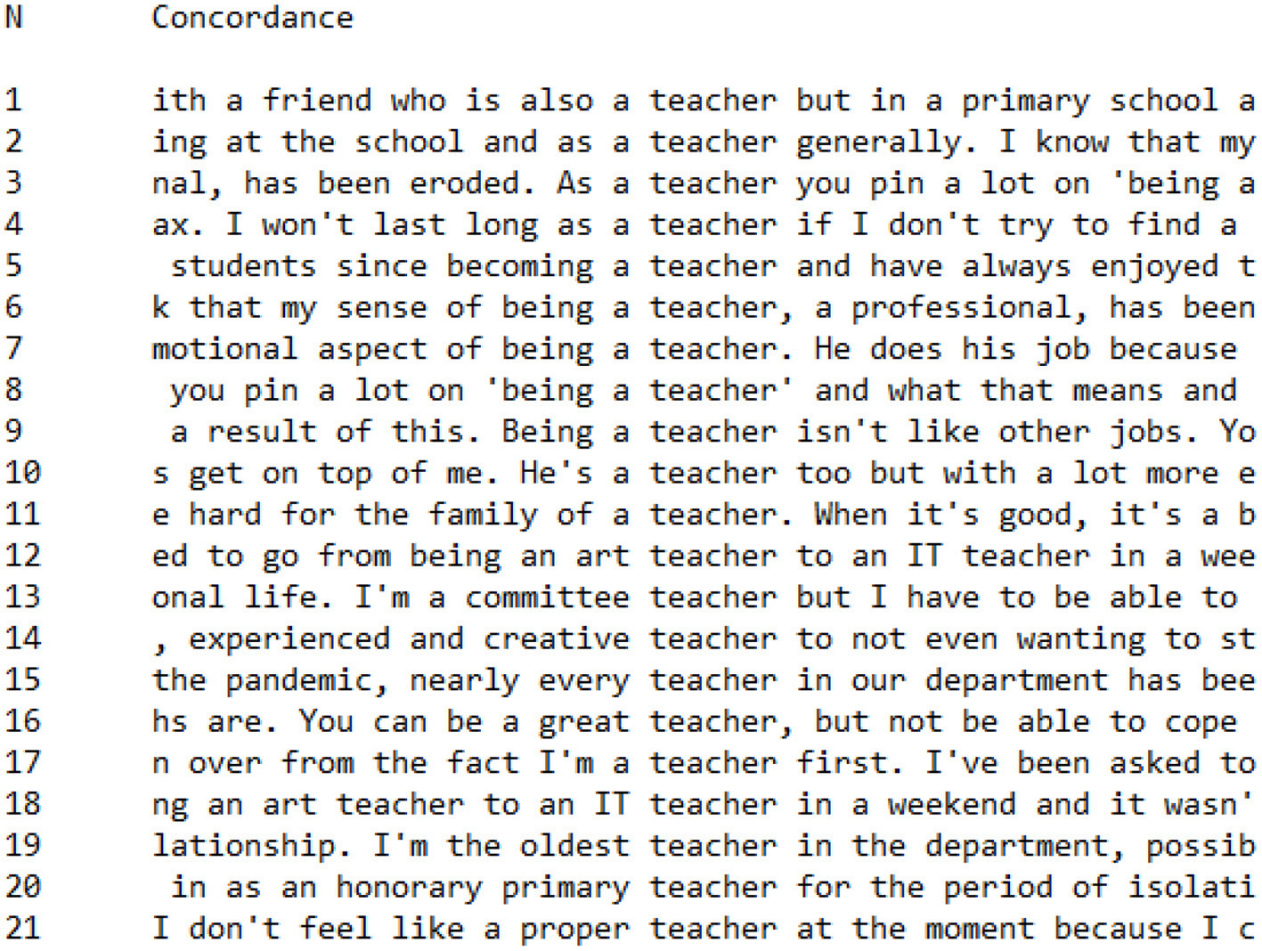
Concordance showing utterances of *teacher* in PI Group interviews.

In order to explore how participants constructed their identities as teachers, concordances for the word *teacher* were analyzed across the CI Group (Figure 5) and PI Group (Figure 6). We hoped that such an analysis would provide a way in to exploring the ways in which teachers described their roles and characteristics during the Covid-19 pandemic. When comparing concordances of *teacher* across the CI Group and PI Group, it became evident that there was a significant quantitative difference between the two groups. There were 21 uses of the word *teacher* within the PI Group interviews, yet only seven in the CI Group interviews. This disparity suggests that for PI Group participants, the subject of the teacher was an object of discourse (Fairclough, 1992); the frequent use of the term *teacher* suggests that the role and characteristics of the teacher are being discursively constituted and renewed, rather than being accepted. PI Group participants expended significantly more time focusing on the teacher than their CI Group counterparts, because CI Group teachers were not as focused on working discursively through what being a teacher meant during the Covid-19 pandemic period of remote teaching.

When the concordances are analyzed qualitatively in more detail, this distinction between CI Group and PI Group teachers becomes even more apparent. Figure 5 is the concordance showing references to *teacher* within the CI Group sub-corpus. In one of these utterances, there does appear to be some performative effort made to construct a teacher identity:

Being a **teacher** means that you give up

In another utterance, the participant explicitly refers to their “identity as a teacher and leader,” which again has a performative effect. However, in the other five utterances, the word *teacher* appears to be deployed in a descriptive capacity rather than a performative one, as in:

the other **teacher** needed to be in bed
assessed to the class **teacher**
rallied round the **teacher** we want to praise
like to be with the same **teacher** all the time

In these utterances, the intention of the sentence is primarily to report incidents or school policies, rather than to rhetorically position teachers and teaching.

In contrast, there were repeated utterances within PI Group interviews to teacher identity, indicating discursive attempts to make sense of or rhetorically justify teacher identity. This was most prominent in the repeated collocation *being a teacher* (*n* = 4):

my sense of **being a teacher**
emotional aspect of **being a teacher**
you pin a lot on **“being a teacher”**
**Being a teacher** isn't like other jobs

These utterances indicate that participants are discursively working through changes in their professional role and identity, rhetorically justifying their actions, and feelings. Rather than talking as if the role of the teacher is accepted and understood, as with the CI Group, PI Group teachers foreground the challenges they face in making sense of their identity and what it means to “be a teacher” during the Covid-19 crisis.

In other utterances, PI Group teachers explicitly position themselves as a certain “type” of teacher:

I'm a **committee teacher**
**experienced and creative teacher**
**oldest teacher** in the department

Finally, in a number of utterances, the identity work brought about by the Covid-19 pandemic lockdown is explicitly discussed by PI Group teachers:

an **art teacher** to an **IT teacher** in a weekend
**honorary primary teacher** for the period of isolation

In these statements, PI Group participants attempt to make sense of the shifts in identity caused by the requirement to teach remotely. It is significant that there are no such statements within the CI Group data. For CI Group participants, relative lack of discussion about what it means to be a teacher indicates a stable and consistent sense of teacher identity. In contrast, PI Group participants talk about teachers more because the Covid-19 pandemic has caused them to navigate changes to their professional identity, destabilizing their sense of professional self.

##### Feelings and Stress

**Figure 7:**
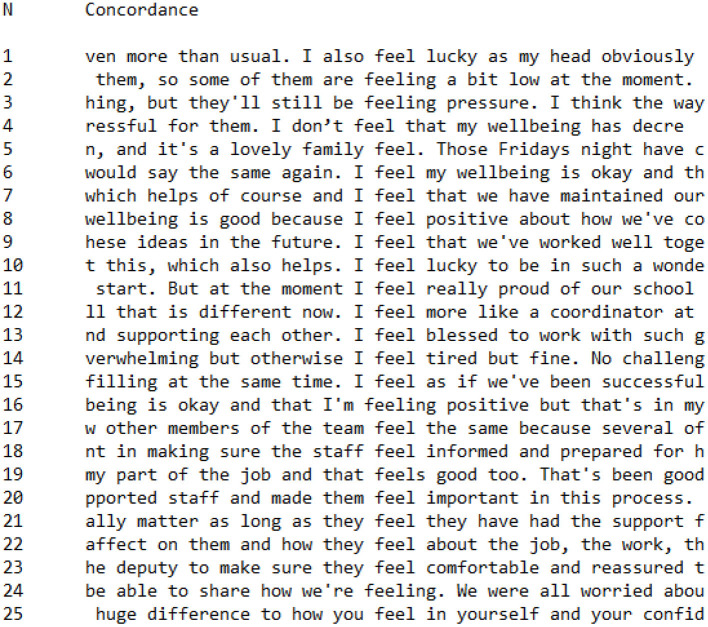
Concordance showing utterances of feel* in CI Group interviews.

**Figure 8:**
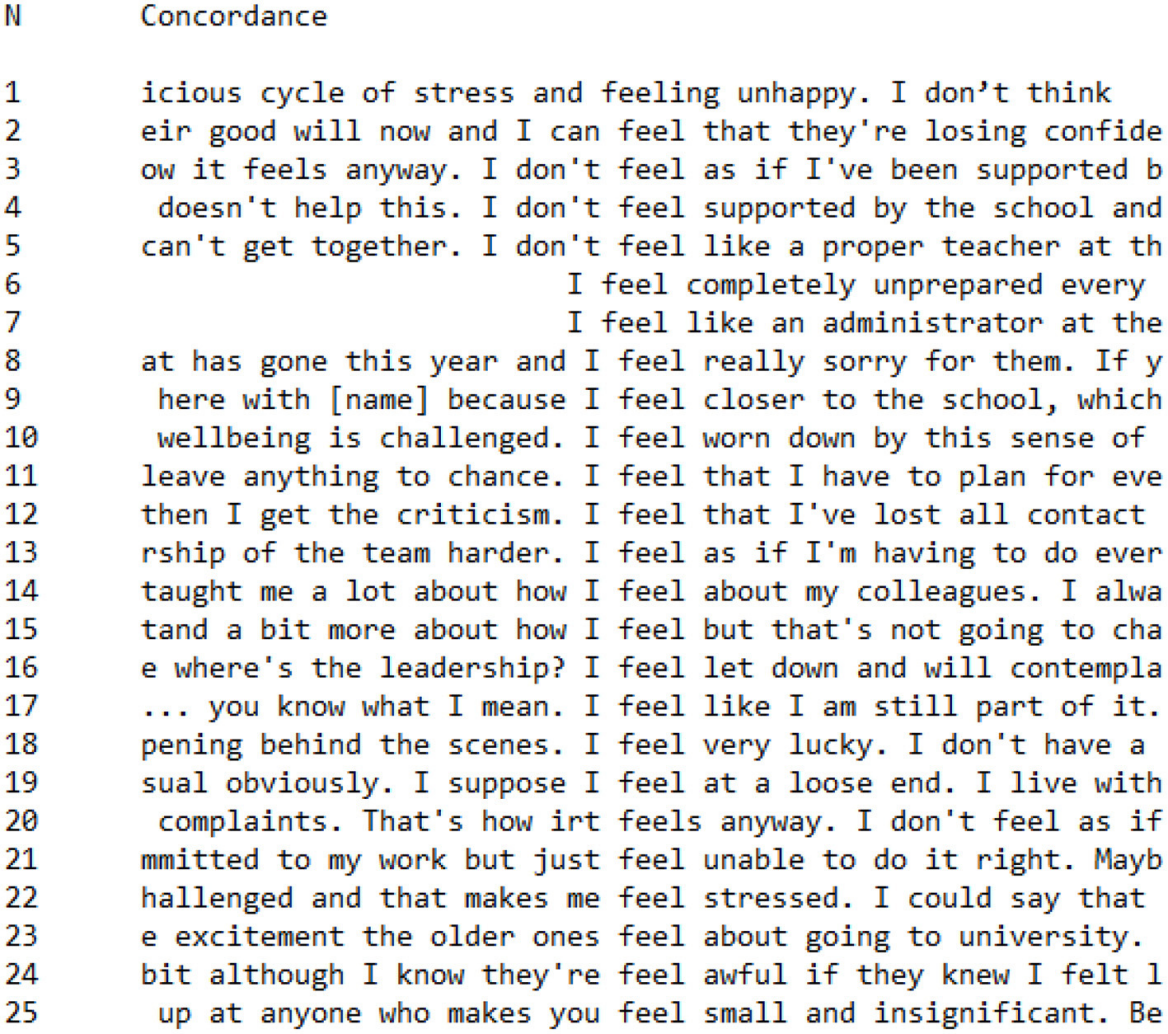
Concordance showing utterances of feel* in PI Group interviews.

There was a clear distinction in the way that CI Group and PI Group participants constructed their feelings, as indicated by the concordances shown in Figure 7 (CI Group) and Figure 8 (PI Group). CI Group members were significantly more likely than PI Group members to associate positive emotions with the word *feel* than negative:

I also **feel lucky**
I **feel positive**
I **feel blessed**
that **feels good**
I **feel** really **proud**
make sure they **feel comfortable**
it's a **lovely family feel**

In total, within the CI Group, 16 of the 25 references to feel^*^ associated this word with positive emotions or processes. These findings support the discourse analysis which identified CI Group participants as constructing a positive perspective on the Covid-19 pandemic lockdown.

In contrast, utterances from members of the PI Group had a tendency to associate negative emotions with the word *feel*, as in the following examples:

I **feel worn down**
I **feel** that I've **lost** all contact
I **feel let down**
I **feel** at a **loose end**
I can **feel** that they're **losing confidence**
I **don't feel supported**

Out of 25 occurrences of the word feel^*^ within the corpus of PI Group speeches, 17 were associated with negative emotions or processes. Again, this finding supports the discourse analysis which indicated that PI Group participants generally constructed a negative perspective on the Covid-19 pandemic in comparison to their CI Group counterparts.

**Figure 9:**
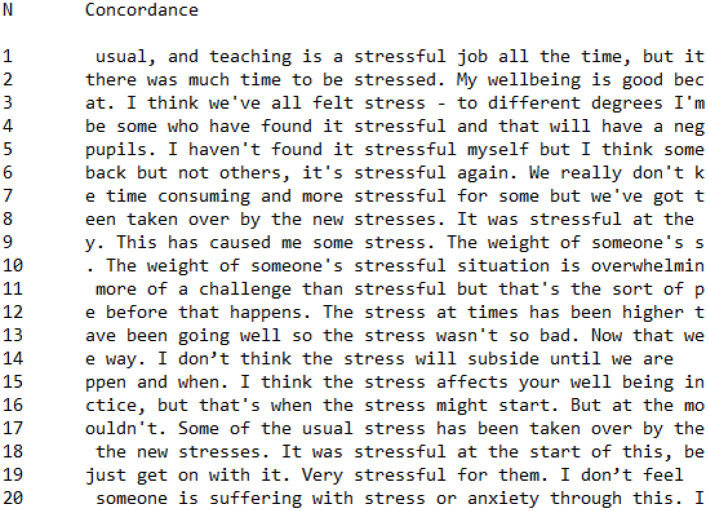
Concordance showing utterances of stress* in CI Group interviews.

**Figure 10:**
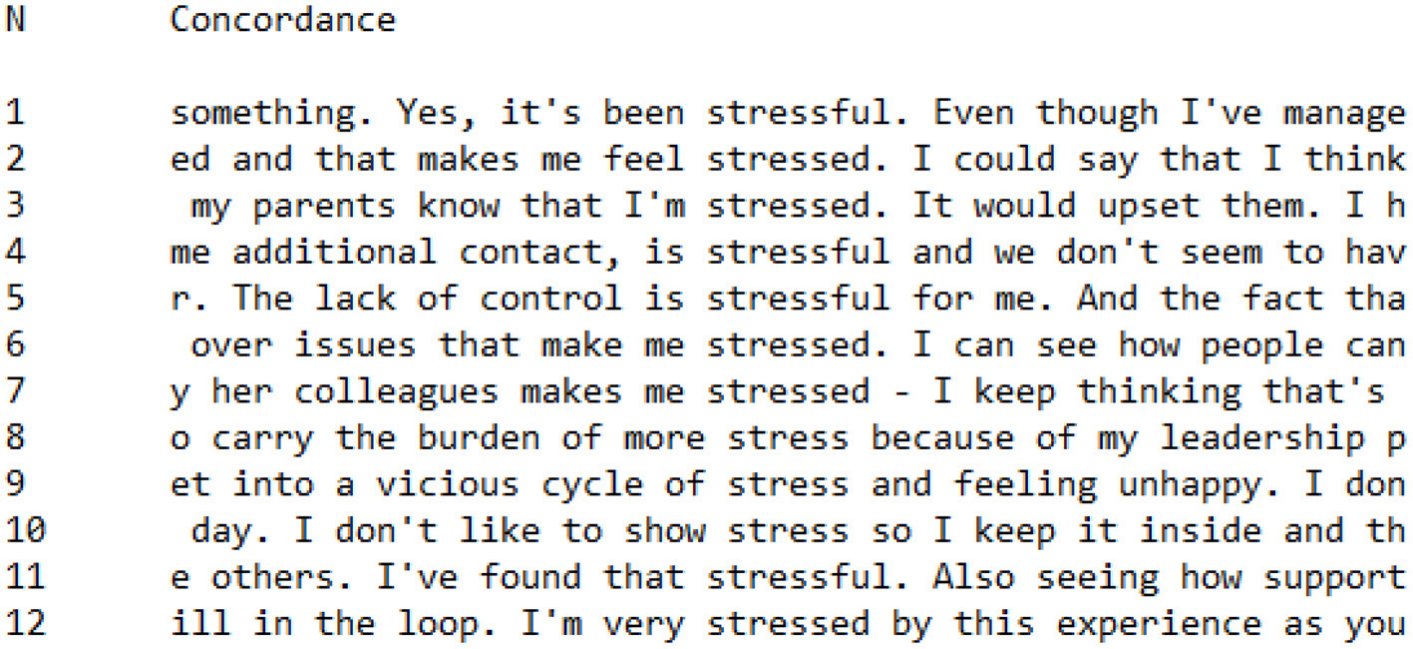
Concordance showing utterances of stress* in PI Group interviews.

In addition to analyzing data related to feelings which was prompted by wordlist data, we also chose to analyze linguistic data specifically regarding the use of the term *stress* across CI and PI Groups^3^. Figures 9, 10 are concordances showing every occurrence of words with the root stress^*^ (*stress, stressful, stress*) across CI (Figure 8) and PI (Figure 9) Groups. It is interesting to note that there were more occurrences of stress^*^ in the CI Group (*n* = 20) than the PI Group (*n* = 12), despite the CI Group having a more positive perspective on the Covid-19 pandemic. Quantitative data alone would therefore indicate that members of the CI Group were more concerned with stress than members of the PI Group. However, when these utterances of stress^*^ were contextualized using concordance data, a pattern became clear in the way that CI Group and PI Group members conceptualized stress.

Looking at the PI Group concordance, (Figure 10), the first person pronouns *me* (*n* = 4) and *I* (*n* = 4) feature heavily in close proximity to stress^*^, for example: Tamara stated in her interview that:

The lack of control is **stressful** for **me**
make(s) **me stressed**.
**I** don't like to show **stress** so **I** keep it inside.
**I've** found that **stressful**
**I'm** very **stressed** by this experience

Members of the PI Group had a tendency to focus on the impact of stress on themselves as individuals, foregrounding the outcome of stress on their internal psychological state.

In comparison, the most common collocations with stress^*^ in the CI group were *it* (*n* = 4) and *the* (*n* = 6). Examples of *it* in a close relationship with stress^*^ include:

I haven't found **it stressful** myself
**it's stressful** again
some who have found **it stressful**.

In these statements, stress is most closely associated with the situation of teaching during the Covid-19 pandemic, rather than being constructed as causing a psychological effect within the individual speaking. Examples of *the* in a close relationship with *stress* within CI Group utterances include:

**the** new **stresses**
that's when **the stress** might start
**the stress** at times has been higher

In these utterances, stress is constructed as something external to the person speaking. Stress in these utterances may indeed be constructed as having a psychological impact, but the use of *the* makes the sense of stress more general, affecting teachers or people generally rather than the individual specifically. In the case of the utterance “I think the stress affects your well-being,” the shift of footing (Goffman, 1981) from *I* to *your* indicates an attempt to generalize the experience of stress during the Covid-19 pandemic, as the interviewee attempted to build common ground between herself and the interviewer by assuming a common experience of stress.

In a number of utterances, CI Group participants explicitly referred to the stress of others:

Very **stressful** for **them**
The weight of **someone's stressful** situation is overwhelming.
**some** who have found it **stressful**
**we've** all felt **stress**.

In these utterances, CI Group members emphasized the communal feeling of stress that affected their social group, in contrast to PI Group utterances which foregrounded the impact of stress upon themselves as individuals.

The salience of either personal or collective identity appears, therefore, to be predictive of how individuals construct stress. Participants in the CI Group had a tendency to construct stress as external to themselves and, in repeated occurrences, portrayed concerns about the stress of others. In contrast, participants in the PI Group generally emphasized their personal experiences of stress, constructing stress as having a detrimental impact on them specifically. Although there a small number of deviant cases were identified, overall there was a clear pattern which distinguished the way that CI Group and PI Group members constructed stress during the Covid-19 pandemic.

### Discursive Justifications

The aim of conducting a discourse analysis within the field of discursive psychology is to take a “functionally oriented approach to the analysis of talk and text” (Edwards and Potter, 1992, p. 27). As such, analysis should not simply be descriptive, but should seek to make sense of the discursive justifications that people use to explain their feelings and behavior. In the final section of the analysis, we returned to the interview transcripts to understand *why* teachers constructed their identities in particular ways—as collective in the CI Group, and personal in the case of the PI Group.

For teachers in the PI Group, the construction of a salient personal identity served as a justification for a loss of commitment and motivation:

*I've been out in a really awful position. My team are looking to me for guidance but I don't know what we're supposed to be doing. I'm losing their good will now and I can feel that they're losing confidence in me. Although we're a relatively big school, we're in the middle of a close city community and I'm not sure how I'll be able to go back at this rate*. (Timothy)

*I feel let down and will contemplate my position over the summer. I won't move to another school*—*it's too late for me to do that, but I don't have to go on teaching if I don't want to. I've been doing this for over 40 years so I have a choice to make*. (Christopher)

*Rubbish, rubbish, rubbish! One headteacher responsible for reducing this committed, experienced and creative teacher to not even wanting to stay in the profession!* (Matilda)

*I also need to make sure I have some kind of life outside work because I worry about it too much and never really relax. I won't last long as a teacher if I don't try to find a better balance...and I love my job so that's a big thing to say*. (Ava)

Teachers in the PI Group emphasized their personal identity to work through difficult feelings about being a teacher, and to justify changes in their teacher identity. For Matilda, emphasizing her personal identity provided some justification for her identity shift from a “committed, experienced and creative teacher” to someone who wanted to leave the profession. Timothy emphasized how he felt isolated from his team and used this to explain how he would find it difficult to return to his school. Christopher foregrounded his personal feelings and his identity as an experienced teacher to justify his decision to “contemplate my position over summer.” Ava, although keen to stress that she enjoyed teaching by saying “I love my job,” argued that she needed to “have some kind of life outside work,” rhetorically using a desire for a sense of identity outside work as a justification for concerns about a future lack of motivation and commitment. For the PI Group, constructing their identities as distinct from their school community provided a justification for the negative admission that they were considering leaving teaching as a result of the changes brought about by the Covid-19 pandemic lockdown.

For teachers in the CI Group, the construction of a salient collective identity served to position them as good leaders and managers:

*I've felt more like an army general for the past three months, than I have a headteacher. It's been full on, all hands to the pump, but we've pulled through it and I think we'll be stronger for it*. (Edwin)

*I've taken it head on and done everything needed to take the staff with me. We're a unit and we had to tackle this as a unit. This was the biggest challenge we had faced as a team so we all had to be on board with the decisions made. I've had to hand over all of my actual teaching. I think it's important that the deputy head teaches, but in this situation, that wasn't possible. I'm really sad about it, but we all had to make sacrifices and that was mine*. (Ivy)

*It's been an interesting experience and one which has brought us altogether in many ways. My role as a leader in the school has been important in making sure the staff feel informed and prepared for how we move forward both during and after the closure. As deputy, I've been responsible in implementing the remote teaching strategy across the school, but with the help of the Key stage leads and subject coordinators*. (Isaac)

*I'm Head of Science and I usually work very closely with the leads for the three sciences and we haven't been able to do that in the same way as before. The departmental meetings have been done differently, as has planning and assessment*. (Maria)

*I'm taking on the role of the head really which he does the bigger planning of how to move forward when the children can come back to school. Our roles have changed a lot, but it's worked and we're really proud that our small school has coped well with it all*. (Noah)

As with teachers in the PI Group, those in the CI Group also reported undergoing changes to their role as a result of the Covid-19 crisis. This rhetorical argument is perhaps most obvious in Edwin's dialogue. He starts by comparing his changed role under Covid-19 to one of an “army general,” but then constructs a sense of democratic and consensual leadership by using the pronoun we: “we've pulled through it […] we'll be stronger for it.” Edwin therefore justifies his changed role by emphasizing a sense of social identity within his school institution. Similarly, Ivy and Noah reported significant changes to their role, but both justified these changes by emphasizing that they were part of a wider group of teachers within their school, all of whom had experienced changes to their role. Maria emphasized her democratic approach to leadership prior to the Covid-19 crisis, and Isaac similarly foregrounded an inclusive leadership style in response to the Covid-19 pandemic. By emphasizing their shared experience with other teachers, rather than their distinctiveness and individual experience, teachers in the CI Group discursively justified the decisions they had made during the period of remote teaching.

It is interesting that all teachers in the CI Group explicitly categorized themselves as having senior leadership roles: Edwin as a headteacher; Ivy, Isaac, and Noah as deputy heads; and Maria as head of department. Findings suggest that the discursive construction of a social identity and portrayal of a democratic, inclusive leadership style were used to justify rapid changes to school structure and policies during the Covid-19 crisis. Members of the CI Group detail changes to their role, but justify these changes as being supported by their staff and as being aligned with the experiences of other teachers within their schools.

## Discussion

Previous research published on education during the Covid-19 crisis has highlighted changes in teacher–pupil relationships which occurred as a result of the sudden requirement to teach remotely (Jones and Kessler, 2020; Moss et al., 2020; Wong, 2020). Our research has extended this body of knowledge by exploring the way that teachers spoke about their relationships with other *teachers* during this challenging time. Research on teacher–pupil relationships highlights how teachers' sense of professional identity shifted as welfare support for children took priority, with teachers organizing food banks and delivering learning materials (Moss et al., 2020). The requirement to teach online was particularly challenging for those who sought to construct respectful and communicative relationships with families and children embedded within an ethic of care (Jones and Kessler, 2020; Ferguson et al., 2021), and for teachers who sought to meet children's basic need for relatedness (Wong, 2020). Our research on teachers' relationships with their colleagues appears to suggest that it was not only relationships with *students* which demanded identity work during the Covid-19 crisis, but also relationships with other teachers.

Our research indicates that teachers who presented a salient collective identity, emphasizing strong and positive relationships between staff and a sense of belonging, also constructed a more positive perspective on the Covid-19 crisis than teachers who presented a salient personal identity. This finding supports the work of Day et al. (2007) which found that teachers who had unstable professional and situated identities were more vulnerable than teachers whose identities were in balance. Although all teachers during the period of Covid-19 suffered from instability in their professional identity as remote teaching was implemented and their professional role changed, teachers in the CI Group appeared to maintain a more stable situated identity than those in the PI Group, reporting more consistent and positive relationships with colleagues. Teachers in the PI Group constructed both their professional and situated identities as being unstable during the Covid-19 crisis, and it is perhaps therefore unsurprising that the PI Group reported a lack of commitment, motivation and resilience during the Covid-19 lockdown.

In line with much research which indicates the protective effects of social support and collective identity both in school settings and elsewhere (Kinman et al., 2011; Drury, 2012; Jetten et al., 2017), our research suggests that teachers who presented themselves as being supported by other teachers within their school may have felt more able to cope with the challenges presented by the Covid-19 lockdown. Certainly, our research has indicated a close association between discursive constructions of collective and personal identity, perspectives on the pandemic, and psychological issues including stress. Like the teachers in Kim and Asbury's (2020) study, the CI Group of teachers within our study constructed a strong sense of shared or collective identity which they argued enabled them to navigate the difficulties of the Covid-19 pandemic lockdown. However, our study also revealed another group of teachers, as represented by the PI Group, who constructed themselves as lacking social support and, consequently, as feeling extremely vulnerable as a result of the Covid-19 crisis. Our study therefore challenges one of the claims of Kim and Asbury's study, that teachers “made extra efforts to create and develop relationships with each other” (2020, p. 1077) during the Covid-19 lockdown. Whereas, this may have been true of teachers who constructed a salient collective identity, other teachers who constructed a salient personal identity reported feeling isolated from their peers, and making efforts to distance themselves by considering leaving the profession.

It was interesting to note that despite previous research showing that female teachers were more likely to deploy functional coping strategies (such as seeking social support) than male teachers (Klapproth et al., 2020; Truzoli et al., 2021), our project indicated no significant difference between the way that male and female teachers spoke about their construction of social identity and use of social support. The majority of teachers in the CI Group (three of five) were male, and the majority of teachers in the PI Group were female (again, three out of five), suggesting that male teachers were more likely to seek out social support than females. This finding could be a function of the small sample size and requires further investigation. However, it may also point to the importance of research which departs from individuals' own identity constructs, rather than from assuming the priority of predetermined groups such as gender.

In terms of the way that participants categorized themselves, one significant difference between the CI Group and the PI Group was the self-categorization of CI Group teachers as senior leaders within their schools. All of the CI Group categorized themselves as senior leaders and used a construction of social identity to present their “selves” as effective managers during this time of difficulty. Our research findings therefore have an interesting relationship to those of Ferguson et al. who, in their research with primary head teachers during the Covid-19 lockdown in Scotland, found that “Head Teachers demonstrated indomitable attentiveness, responsiveness, and responsibility for others, thus showing that relationships are fundamentally about values within education” (2021, p. 11). The findings from our project highlight that one of the rhetorical devices employed by headteachers and other senior leaders to justify their actions and professional identity during the Covid-19 crisis was to emphasize collegial relationships with others. Our research does not contradict the findings of Ferguson et al. (2021); in many ways the findings of the two studies are aligned. However, our findings emphasize the importance of attending to the rhetorical *purpose* of such claims and their function in discourse, rather than accepting such self-positionings as representative of an objective reality.

With regard particularly to PI Group teachers, our findings support the work of previous research on stress within the field of critical education studies and discursive psychology. Teachers in the PI Group constructed a salient personal identity partially to justify their feelings of stress, shifting the locus of responsibility for such stress from themselves and onto others. This supports Kelly and Colquhoun's argument that prominent psychological discourses within school settings not only encourage teachers to view themselves as stressed, but as “responsible for managing that stress” (2003, p. 202). In order to rhetorically manage the negative feelings associated with being unable to manage the stress brought about by the Covid-19 lockdown, PI Group teachers emphasized negative relationships with peers. The findings from the present study extend the findings of Hepburn and Brown (2001), who found that teachers use the discourse of stress to protect themselves from accusations. We found that teachers also use discourses oriented around negative relationships with peers to justify their negative feelings and future actions, particularly those associated with attrition; as such, our findings support those of Thomson (2008) who detailed how a headteacher used discourses of stress to justify decisions to leave the profession.

## Conclusion

This paper has made contributed to the growing field of study concerning the impact of Covid-19 lockdowns on individuals and social groups. We have argued that interpersonal pronoun usage may serve as a predictor of teachers' perspectives on the Covid-19 crisis, extending previous research on the impact of teachers' peer relationships during this unprecedented time by employing a methodological stance informed by discursive psychology. This paper does not seek to claim that interpersonal pronoun use is sufficient to explain and understand teacher identities in their entirety, either during the Covid-19 crisis or during other challenging situations. More research would need to be conducted with a larger sample in order to determine whether the findings of this study are generalizable within a wider and more representative teaching population, or during other times of stress or difficulty. Although it is usual for studies within the field of discursive psychology to rely on interview data from a small sample, innovative use of corpus-assisted discourse analysis (as employed in this paper) could enable future studies to work with larger samples to determine the generalizability of the results presented here. Therefore, although one of the limitations of this study was the small sample size, the study could serve as a pilot for future work exploring teachers' discursive constructions of peer relationships.

## Data Availability Statement

The raw data supporting the conclusions of this article will be made available by the authors, without undue reservation.

## Ethics Statement

This research study was reviewed and approved by University of Worcester Arts, Humanities and Education Research Ethics Panel. The participants provided their written informed consent to participate in this study.

## Author Contributions

AK was responsible for the development of the research project and the collection of data. AK, KS, and MW completed initial thematic analysis. KS completed discourse analysis and wrote the findings, discussion, and conclusion. MW and KS developed the conceptual framework and wrote the introduction. AK and KS wrote the methodology, revised, and edited the manuscript together before submission. All authors contributed to the article and approved the submitted version.

## Conflict of Interest

The authors declare that the research was conducted in the absence of any commercial or financial relationships that could be construed as a potential conflict of interest.

## Publisher's Note

All claims expressed in this article are solely those of the authors and do not necessarily represent those of their affiliated organizations, or those of the publisher, the editors and the reviewers. Any product that may be evaluated in this article, or claim that may be made by its manufacturer, is not guaranteed or endorsed by the publisher.

## Footnotes

^1^The career phase groupings for this study were selected based on research conducted by Day et al. (2007), who identified six phases reflecting variations in teachers' identity, commitment, and self-efficacy. These phases were: 0–3 years; 4–7 years; 8–15 years; 16–23 years; 24–30 years; and 31+ years.

^2^Content words contribute to the meaning of a sentence, rather than the grammar.

^3^*Stress* was not the only word which was analyzed outside the high frequency content words identified in Table 3, but the findings concerning stress were particularly relevant and therefore warranted inclusion within this paper.
